# Feasibility, reliability and validity of a modified approach to goal attainment scaling to measure goal outcomes following cognitive remediation in a residential substance use disorder rehabilitation setting

**DOI:** 10.1080/00049530.2023.2170652

**Published:** 2023-02-19

**Authors:** Jamie Berry, Ely M. Marceau, Jo Lunn

**Affiliations:** a Department of Psychology, Macquarie University, Sydney, NSW, Australia; b Advanced Neuropsychological Treatment Services, Strathfield South, NSW, Australia; c School of Psychology and Illawarra Health and Medical Research Institute, University of Wollongong, Wollongong, NSW, Australia; d We Help Ourselves (WHOs), Lilyfield, NSW, Australia

**Keywords:** Goal attainment scaling, GAS, goal setting, substance use disorder, cognitive remediation

## Abstract

**Objective:**

Although person-centred outcome measures have been recommended to evaluate cognitive rehabilitation interventions, few validated measures have been developed for this purpose. The current study examined aspects of feasibility, reliability and validity of a modified version of goal attainment scaling that uses a goal menu, calculator and control goals.

**Method:**

Participants were N=25 female residents of a substance use disorder therapeutic community who were allocated to a four-week cognitive remediation (n=13) or treatment as usual (n=12) control group in a controlled sequential groups trial. Modified goal attainment scaling was used to set goals. Limited efficacy and efficiency, quality appraisal criteria, and convergent and discriminant validity of target and control goals were used to examine feasibility, reliability and content validity, and construct validity, respectively.

**Results:**

Target goals were achieved at a higher rate than control goals for the Intervention, but not Control, group, with a medium effect size (r = 0.5). The approach was efficient and 44% of reliability and 75% of content validity criteria were met. Target goals correlated more strongly than control goals with the Behavior Rating Inventory of Executive Function - Adult version.

**Conclusions:**

The modified approach to goal attainment scaling demonstrated aspects of feasibility, reliability and validity.

## Key points

**What is already known about this topic:**

1. Cognitive remediation is a promising intervention for people with substance use disorder.
2. Goal attainment scaling captures individualised person-centred goals.
3. There is much variability in the quality and application of goal attainment scaling.

**What this topic adds:**

1. Modified goal attainment scaling is feasible in substance use disorder treatment research.
2. Modified goal attainment scaling meets several reliability and validity criteria.
3. Modified goal attainment scaling can be used to generate an effect size using nonparametric techniques.

## Introduction

Decades of research has focused on the question of whether and how cognitive functioning may be improved following acquired brain injury (Cicerone et al., 2000, 2005, 2011, 2019; Ponsford et al., 2014; Tate et al., 2014; Togher et al., 2014; Velikonja et al., 2014), and more recently substance use disorder (Nardo et al., 2022). Although this research has commonly focused on changes in scores on cognitive tests or standardised questionnaires following a course of intervention, much less attention has been paid to whether person-centred goals are achieved post-intervention.

A recent systematic review concluded that although cognitive remediation is a promising approach for improving cognition and treatment outcomes for people with substance use disorders, there was considerable heterogeneity in the types of interventions, participant characteristics and outcome measures (Nardo et al., 2022). The outcome measures in the reviewed studies could be classified as being either performance-based (i.e., cognitive tests) or inventory-based (i.e., questionnaires). None of the studies utilised goal setting approaches to evaluate whether the interventions resulted in individual goal attainment pertaining to everyday functioning (i.e., ecological goals) despite multiple systematic reviews of evidence-based cognitive rehabilitation for acquired brain injury emphasising the importance of developing and utilising measures of everyday real-world functioning (Cicerone et al., 2000, 2005, 2011, 2019).

### Goal attainment scaling

Goal Attainment Scaling (GAS) was developed more than half a century ago to measure unique and individualised goal outcomes for clients of outpatient mental health services (Kiresuk & Sherman, 1968). Individualised goals that are difficult to capture using standardised measures are set and each goal is scaled so that a range of post-intervention goal outcomes are represented across five levels. The levels are assigned scores of −2 (representing a much worse than expected outcome), −1 (representing a worse than expected outcome), 0 (representing the expected outcome), +1 (representing a better than expected outcome) and+2 (representing a much better than expected outcome). Typically, the post-intervention GAS outcome scores are entered into a formula and a T-score is calculated, which summarises the outcomes for an individual across all their goals (Kiresuk & Sherman, 1968).

In their proposed criteria for evaluating GAS scales as outcome measures in rehabilitation research, Krasny-Pacini et al. (2016) reviewed the major criticisms that GAS methodology has attracted, being: unknown clinimetric qualities due to the idiosyncratic nature of GAS (Steenbeek et al., 2007); subjective scoring; risk of choosing goals that are clinically irrelevant or too easy or challenging to achieve (Ruble et al., 2012); the ordinal nature of the scales with a lack of equidistance between GAS levels (Tennant, 2007; Turner-Stokes et al., 2010); and inappropriate use of T-scores with subjective values (MacKay et al., 1996; Malec, 1999; Schlosser, 2004).

Grant et al. (2012) found that Goal Management Training (Levine et al., 2011) resulted in sustained improvements on a range of daily activities among individuals with severe traumatic brain injury using GAS. However, they noted several practical limitations of using GAS, including: identifying appropriate goals for each participant; breaking down large goals into subgoals; and breaking down subgoals into five GAS levels. A comprehensive critique of the GAS methodology adopted in that study was subsequently undertaken by two of the authors, and recommendations were made to address GAS methodology limitations, including: having only one variable per GAS scale; considering all possible outcomes; defining all five GAS levels; ensuring that all five GAS levels are mutually exclusive; ensuring that all goals are mutually exclusive; and ensuring there are no gaps between GAS levels (Grant & Ponsford, 2014). The length of time taken to set and scale the goals was also problematic, with 2–4 hours required to set and scale three goals (Grant & Ponsford, 2014; Grant et al., 2012).

### The current study

The current research aimed to address many of the quality appraisal criteria of Krasny-Pacini et al. (2016) and the practical GAS scale construction difficulties noted by Grant and Ponsford (2014) by applying a novel modified version of GAS in evaluating individualised goal outcomes following cognitive remediation offered to residents of an SUD treatment program. Marceau et al. (2017) previously showed that a 12-session cognitive remediation program resulted in improvements in inhibition (Stroop test; Golden & Freshwater, 2002) and self-reported impulsivity (Barratt Impulsiveness Scale; Patton et al., 1995), self-control (Brief Self-Control Scale; Maloney et al., 2012) and executive functions (Behavior Rating Inventory of Executive Function – Adult version – BRIEF-A; Roth et al., 2005) compared to a treatment as usual control condition in a female-only therapeutic community. By way of extending these findings, a purpose of the current study was to examine whether modified GAS was also sensitive to the intervention, hence demonstrating convergent validity.

Setting both control and target goals allows each individual to act as their own control, and hence allows for the calculation of an effect of target to control goal attainment for each individual. Whilst this approach was adopted in an evaluation of physical therapy outcomes for individuals with severely limited physical and cognitive abilities (Brown et al., 1998), the effect size was inappropriate as it utilised GAS T-scores rather than non-parametric methods. We addressed this limitation by applying non-parametric analyses in the present study.

The general hypotheses were that the modified approach would be feasible according to two of Bowen et al’.s (2009) feasibility criteria: i) limited efficacy (i.e., that calculation of an effect size of target to control goals was possible) and ii) practicality (i.e., that the approach would be efficient). It was also predicted that the approach would demonstrate reliability and validity according to the Krasny-Pacini et al. (2016) quality criteria and that construct validity would be demonstrated in relation to a standardised self-report inventory of executive functioning.

The specific hypotheses were that: i) participants in the intervention group would attain their target goals at a higher rate than their control goals, whereas those in the control group would have equal target and control goal attainment; ii) goal selection and scaling would be more efficient than that described in Grant and Ponsford (2014); iii) the majority of the Krasny-Pacini et al. (2016) GAS quality appraisal criteria for reliability and content validity would be met, and iv) there would be a stronger correlation between the BRIEF-A (Roth et al., 2005) and target goals than control goals.

## Method

### Participants

Participants were *N* = 25 residents of a female-only residential SUD therapeutic community in Sydney, Australia. Inclusion criteria for the study were: (i) diagnosis of SUD (a condition of entry into the rehabilitation facility, which was confirmed using the Mini-International Neuropsychiatric Interview – MINI-Plus; Sheehan et al., 1998), (ii) a minimum abstinence period of 7 days (with confirmation of detoxification), (iii) absence of any self-reported neurological, infectious, or other disease affecting the central nervous system except for traumatic brain injury due to the high prevalence of traumatic brain injury in residential SUD rehabilitation facilities (Marceau et al., 2016), (iv) English as a first language and (v) GAS data available at four-month post-intervention follow-up. A condition of staying at the residential facility was that participants remained abstinent from substances of misuse.

### Materials

#### Goal menu

A goal menu comprising 20 everyday self-control and executive functioning behaviours was developed (see Supplement 1). Items reflected healthy daily habits (e.g., to eat healthier food), impulse control (e.g., to control my temper or emotions), organisation (e.g., to be able to find things quickly and easily), initiative (e.g., to do things right away), persistence (e.g., to see things through to completion), flexibility (e.g., to respond better to change) and memory/attention (e.g., to concentrate better whilst ______).

#### Maximum realistic level and current functioning questions

For each of the 20 goal menu items, a maximum realistic level (MRL) and current functioning (CF) question example was provided to guide the examiner when setting goals with the participant (see Supplement 2).

### Procedure

Ethics approval to conduct this study was granted by the University of Wollongong and Illawarra and Shoalhaven Local Health District Health and Medical Human Research Ethics Committee (approval number HE15/206).

#### Study design

The study was a controlled sequential groups trial, with recruitment commencing in July 2015. After providing consent to participate in the research, participants were assigned to either a treatment as usual (Control) or treatment as usual plus cognitive remediation (Intervention) group. All residents of the service at the time of recruitment were invited to participate in the trial, and the participation rate was 96%. The Intervention group was recruited first followed by the Control group, following a washout period when all Intervention participants had exited the program. Participants in the Intervention group attended a total of 12 × two-hour group sessions across 4 weeks (three sessions per week). Each two-hour session comprised a strategy training component (1 hour) and computerised cognitive training component (1 hour). All sessions were facilitated by the first author (JB) and co-facilitated by the second author (EMM) who was also involved in pre- and post-intervention testing.

#### Intervention

##### Strategy training

The group-based cognitive remediation intervention was developed with a strong emphasis on the remediation of executive functions and self-regulation in view of the finding that executive functioning is particularly impaired in an SUD treatment population (Fernández-Serrano et al., 2010; Valls-Serrano et al., 2016). Details regarding the elements and structure of the program are found in Marceau et al. (2017). The facilitators followed a manual to ensure treatment consistency. Participants were required to select any goal of their choosing in order to apply a mental contrasting with implementation intentions exercise in modules eight and nine. Intervention group participants were provided with their target GAS goals to use for this exercise if they wished.

##### Computerised cognitive training

The strategy-based training comprised the first hour of each session. In the second hour, following a short break, participants played specific Lumosity games (Lumosity, 2021) on iPads in a group setting. They were instructed to use and practice the strategies they learnt about in the previous hour of strategy training. After each of three 10–15-min blocks of computerised training, the facilitator asked participants to share with the other group members the strategies they found useful whilst completing the cognitive training exercises.

#### Data collection

All participants completed the GAS goal setting process, together with a battery of cognitive tests and questionnaires (Marceau et al., 2017) at baseline. Post-intervention measures were collected at an average of 4.5 weeks (SD = 0.55) following baseline assessment, allowing a four-week period for the groups to receive treatment. A third assessment (follow-up) including a final GAS outcome measurement was undertaken at an average of 21.2 weeks (SD = 4.14) post-baseline, which was used in the current study because the post-intervention outcomes included a retrospective evaluation period that overlapped with the active intervention or control phase.

### Measures

#### Behavior rating inventory of executive function – adult version (BRIEF-A; Roth et al., 2005)

The BRIEF-A is a 75-item self-report questionnaire consisting of nine subscales. Participants are instructed to answer each question by selecting never, sometimes, or often, in relation to the frequency with which they have had problems with any of the listed behaviours in the previous month. The Global Executive Composite (GEC) provides an overall summary score on a T-distribution, with higher scores indicating more severe impairment.

#### Modified goal attainment scaling

Table 1 outlines instructions for the modified GAS goal setting, scaling and assessment processes as well as a hypothetical example. This approach was based on use of an online calculator that automatically calculated the GAS ranges based on the participants’ current level of functioning and their maximum realistic level of functioning for the chosen goal behaviour, adopted from Clark et al. (2021).

**Table 1.** Modified GAS instructions and a hypothetical example.

Step		Instructions	Hypothetical example
1	Explanation	State: “Over the course of this study, we are interested in knowing whether your memory, thinking and self-regulation skills improve in day-to-day activities”.	E: “Over the course of this study, we are interested in knowing whether your memory, thinking and self-regulation skills improve in day-to-day activities”.
2	Goal selection	Hand the Goal Menu to the participant and explain: “Choose two goals from this goal menu that you would like to work on”	E: “Choose two goals from this goal menu that you would like to work on” P: Chooses goal 15, To remember_____ and specifies: “To remember to bring the things I need with me” (only one goal is exemplified here)
3a	Goal specification	State: “OK, so let’s make those goals really specific to you to make sure we are measuring real changes in your life” and ask about specific real-world examples for each of the selected goals.	E: “OK, so let’s make those goals really specific to you to make sure we are measuring real changes in your life. What is an example of a situation where you need to remember to bring the things you need?”
3b	Goal specification – documentation	Document the responses to questions in 3a for each selected goal	“To bring a pen and notepad to group sessions” is documented
4a	Establishing maximum realistic level	Refer to Maximum Realistic Level and Current Functioning Questions and ask the relevant MRL^d^ question for each selected goal	E: “In a typical week, how many group sessions do you attend?” P: “10”
4b	Maximum realistic level documentation	Document the responses to the questions in 4a	“10” is documented as the MRL^a^
4c	Establishing current functioning	Refer to Maximum Realistic Level and Current Functioning Questions and ask the relevant CF^d^ question for each selected goal	E: “How many times per week do you remember to bring your pen and notepad to groups?” P: “5”
4d	Current functioning documentation	Document the responses to the questions in 4c	“5” is documented as the CF^b^
5	Ensure five levels of measurement	Subtract 4d from 4b. If less than 4, double both 4d and 4b until difference is > = 4. Each time 4b and 4d are doubled, the measurement interval (denominator) should also be doubled. For example, “per week” doubled becomes “per fortnight” and “per fortnight” doubled becomes “per month”	10–5 = 5 (no need to double the values and denominator because 5 > = 4)
6a	Calculation of the GAS^c^ levels/scale	Enter MRL^a^ and CF^b^ values from steps 4b and 4d (if difference is > = 4) or new values from step 5 (with difference > = 4) into the calculator at gas2.com.au	Enter the following values into the online calculator: MRL^a^ = 10, CF^b^ = 5
6b	Ensure goal achievability	Ensure the goal (“expected” outcome from the calculator output) is achievable. If not, reconsider the MRL^a^ and modify accordingly by repeating steps 4 and 5 with a more realistic MRL^a^	The “expected” outcome of remembering to bring a pen and notepad to groups 8 times per week is achievable.
7a	Orientation to the GAS^c^ scale	Show the participant the calculator output on an electronic device or print or transcribe onto paper	+2 much better than expected 10 to 10 +1 better than expected 9 to 9 0 expected 8 to 8 −1 less than expected 7 to 7 −2 much less than expected 4 to 6
7b	Explanation of the GAS^c^ scale	Explain: “So, you’re currently [statement of behaviour] X out of a possible Y times per [interval]. However, it can be hard to motivate oneself to achieve something at the maximum realistic level when one is nowhere near that level currently. So, I suggest the target outcome be in between where you are currently functioning and that maximum realistic outcome. I have made some calculations to show you what I mean. You are currently [statement of behaviour] X times per [interval] and, as we discussed, the maximum realistic level is Y times per [interval]. So, a realistic goal for you might be to [statement of behaviour] Z times per [interval] (pointing to the GAS^c^ = 0 or ‘expected’ range). At the end of the trial we will be able to see whether you have achieved that goal, whether you made progress towards the goal but haven’t achieved it (pointing to the −1 GAS^c^ level), achieved more than the goal (pointing to the+1 GAS^c^ level), made no progress at all (pointing to the −2 GAS^c^ level) or achieved the maximum realistic level (point to the+2 GAS^c^ level)”	E: “So, you’re currently remembering to bring your pen and notepad to groups sessions 5 out of a possible 10 times per week. However, it can be hard to motivate oneself to achieve something at the maximum realistic level when one is nowhere near that level currently. So, I suggest the target outcome be in between where you are currently functioning and that maximum realistic outcome. I have made some calculations to show you what I mean. You are currently bringing your pen and notepad to groups sessions 5 times per week and, as we discussed, the maximum realistic level is 10 times per week. So, a realistic goal for you might be to bring your pen and notepad to groups sessions 8 times per week (pointing to the GAS^c^ = 0 or ‘expected’ range). At the end of the trial we will be able to see whether you have achieved that goal, whether you made progress towards the goal but haven’t achieved it (pointing to the −1 GAS^c^ level: bringing your pen and notepad 7 times per week), achieved more than the goal (pointing to the+1 GAS^c^ level: bringing your pen and notepad 9 times per week), made no progress at all (pointing to the −2 GAS^c^ level: bringing your pen and notepad 4 to 6 times per week) or achieved the maximum realistic level (point to the+2 GAS^c^ level: bringing your pen and notepad 10 times per week)”
8	Documentation of the GAS^c^ scale for target goals	Document the GAS^c^ scale in full, using the specified goal wording, ensuring a time-frame is stipulated. The SMART^d^ goal is represented by the expected outcome (GAS^c^ = 0) statement. This step can be completed later to save time during the goal setting process)	+2 I bring a pen and notepad to group sessions 10 times per week+1 I bring a pen and notepad to group sessions 9 times per week0 I bring a pen and notepad to group sessions 8 times per week-1 I bring a pen and notepad to group sessions 7 times per week-2 I bring a pen and notepad to group sessions 4 to 6 times per week
9a	Establishing maximum realistic level of control goal	Control goals should be set implicitly, so complete this and following steps after some interference task so as to dissociate the following questions from the goal setting process. Randomly choose a non-selected goal on the menu and ask the relevant MRL^a^ question for the chosen goal	Goal 6 on the Goal Menu is randomly chosen by the examiner, and the MRL^a^ question for that goal is asked, E: “In a typical week, how often do you need to be punctual?” P: “7” (only one control goal is exemplified here)
9b	Control goal maximum realistic level documentation	Document the responses to the questions in 9a for each selected control goal	“7” is documented as the MRL^a^
9c	Establishing current functioning of control goal	Refer to Maximum Realistic Level and Current Functioning Questions and ask the relevant CF^b^ question for each selected control goal	E: “How many times per week are you currently punctual?” P: “4”
9d	Control goal current functioning documentation	Document the responses to the questions in 9c for each selected control goal	“4” is documented as the CF^b^
9e	Ensure five levels of measurement of control goal	Subtract 9d from 9b. If less than 4, double both 9d and 9b until difference is > = 4). Each time 9b and 9d are doubled, the measurement interval (denominator) should also be doubled. For example, “per week” doubled becomes “per fortnight” and “per fortnight” doubled becomes “per month”	7–4 = 3. Doubled: 14–8 = 6 (the measurement interval is doubled from “per week” to “per fortnight”)
9f	Calculation of the GAS^c^ levels/scale	Enter MRL^a^ and CF^b^ values from steps 9b and 9d (if difference is > = 4) or new values from step 9e (with difference > = 4) into the calculator at gas2.com.au	MRL^a^ = 14, CF^b^ = 8 (per fortnight)
9 g	Ensure goal achievability	Ensure the goal (“expected” outcome from the calculator output) is achievable. If not, reconsider the MRL^a^ and modify accordingly by repeating steps 4 and 5 with a more realistic MRL^a^	The “expected” outcome of being punctual 11 times per fortnight is achievable.
10	Documentation of the GAS^c^ scale for control goals	Document the GAS^c^ scale in full. This step can be completed later to save time during the goal setting process	+2 I am punctual 14 times per fortnight +1 I am punctual 12 to 13 times per fortnight 0 I am punctual 11 times per fortnight −1 I am punctual 10 times per fortnight −2 I am punctual 7 to 9 times per fortnight
11	Assessment of goal attainment	At the predetermined follow-up interval, ask about the frequency of the target and control goals over the relevant time period	E: “How often did you bring a pen and notepad to group sessions in the past fortnight?”; “How often were you punctual in the past fortnight?”

^a^MRL = maximum realistic level.

^b^CF = current functioning.

^c^GAS = goal attainment scaling.

^d^SMART = specific, measurable, achievable, relevant, time-based.

### Analysis

#### Hypothesis 1: limited efficacy

Two target and two control goals were chosen for each participant using the approach described in Table 1. Notably, although the target goals were explicitly chosen by the participants, the control goals were set implicitly by asking the Maximum Realistic Level and Current Functioning questions pertaining to goal menu items that the examiner randomly selected. Follow-up GAS scores were subtracted from the consistent baseline score of −2 (outcome range 0–4). Although some studies have allowed for the pre-intervention GAS level to be −1, rather than −2 to account for the possibility of deterioration, this limits the range of goal attainment to four, rather than five levels. Ruble et al. (2012) have argued that the use of a consistent −2 baseline is justifiable in populations that are not expected to deteriorate, and maintaining the five-point GAS scale for clinical purposes was supported in a review of GAS in acquired brain injury rehabilitation (Ertzgaard et al., 2011). Applying a consistent −2 baseline also ensures compliance with the recommendation by Krasny-Pacini et al. (2016) for the pre-intervention score to be comparable between groups.

Wilcoxon Signed Rank tests were used to analyse differences between target and control goals (within participants), whereby it was predicted that there would be a significant difference for the Intervention, but not the Control group. A power analysis revealed that a sample size of 10 was required to detect a population mean difference of 1 with a population standard deviation of 1, power of .8 and alpha of .05. The median scores across the two target goals and the two control goals were used in the analyses. The formula for a Pearson r effect size based on Wilcoxon Signed Rank tests (Fritz et al., 2012; Pallant, 2016), $r=\frac{z}{\sqrt{N}}$, was used to calculate the effect of treatment versus control goals for statistically significant differences.

#### Hypothesis 2: practicality

Time taken to set and scale a goal was retrospectively estimated by the examiner to examine practicality, and specifically the efficiency of goal setting and scaling.

#### Hypothesis 3: reliability and content validity

The current study was evaluated against the 17 GAS quality criteria proposed by Krasny-Pacini et al. (2016), which includes items to evaluate i) reliability of scale construction (four items), ii) reliability of scale rating (five items), iii) content validity (four items) and iv) other (four items) criteria. The focus was on whether most of the reliability and content validity criteria were met. To evaluate equidistance of levels, one of the reliability of scale construction criteria, intraclass correlations of the GAS level ranges were calculated for each of the two target and control goals. To fulfil one of the content validity criteria, the target and control goals were classified according to their World Health Organisation International Classification of Health and Disability (ICF; WHO, 2002) domains.

#### Hypothesis 4: construct validity

Bivariate Spearman rank order correlations between BRIEF-A GEC scores and both target and control goal attainment was undertaken to examine construct validity. It was predicted that there would be a higher correlation between BRIEF-A GEC and target goals (convergent validity) than between BRIEF-A GEC scores and control goals (discriminant validity).

## Results

The characteristics of the sample are presented in Table 2.

**Table 2.** Sample characteristics.

Characteristic		Control (n=12)		Intervention (n=13)		Overall (n=25)	
		M	SD	M	SD	M	SD
Age		32.3	8.9	33.7	10.7	33	9.7
Education		11.8	1.6	11.7	2.5	11.8	2.1
TOPF^d^		100.8	12.8	96.9	11.7	98.8	12.1
Years of regular use							
	Alcohol	9.1	5.8	9.8	8.5	9.5	7.2
	Amphetamines^b^	6.8	6.4	6.9	5.9	6.8	6
	Sedatives	3.8	5.8	2.8	5.6	3.3	5.6
	Cannabis	8.1	8	8.1	7.1	8.1	7.4
	Heroin	2	3.6	0	0	1	2.6
	Cocaine	1.4	3.4	1.3	4.4	1.4	3.9
Primary substance of misuse		%		%		%	
	Methamphetamine	25		46.2		36	
	Alcohol	41.7		23.1		32	
	Sedatives	8.3		15.4		12	
	Other Amphetamines	0		15.4		8	
	Cannabis	16.7		0		8	
	Heroin	8.3		0		4	
Loss of consciousness after head injury		66.7		53.8		60	
Hospitalised after head injury		58.3		30.8		44	

^a^TOPF = Test of Premorbid Functioning (Pearson, 2009).

^b^includes methamphetamine.

There were no significant differences between the Control and Intervention groups for age, *t*(23) = −.343, *p* = .735, education, *t*(23) = .165, *p* = .87, estimated premorbid intellect, *t*(23) = .798, *p* = .433, primary substance of misuse, *χ*^*2*^(5) = 6.804, *p* = .236, loss of consciousness following head injury, *χ*^*2*^(1) = .427, *p* = .513, or hospitalisation following head injury, *χ*^*2*^(1) = 1.924, *p* = .165.

The final sample comprised *n* = 12 Control and *n* = 13 Intervention participants. The Control participants set a total of 24 target and 23 control goals and the Intervention participants set a total of 26 target and 23 control goals. However, *n* = 3 Intervention participants and *n* = 1 Control participant had set only one control goal, meaning that median values could not be calculated. Hence, the final analyses were conducted on data from *n* = 11 Control and *n* = 10 Intervention participants. A missing values analysis was conducted using Little’s MCAR test (Little, 1988), revealing non-significant results, *χ*^*2*^(1) *=* .899, *p* = .343, indicating that the data were missing completely at random.

### Hypothesis 1: limited efficacy

Target goal attainment (*Mdn* = 4, 3–4) was greater than control goal attainment (*Mdn* = 2, 1.125–4; *Z* = 2.232, p = .026) for the Intervention group. The effect of target versus control goals for the Intervention group was $\frac{2.232}{\sqrt{20}}$ = .5. There was no difference between target (*Mdn* = 2.5, 2–3) and control (*Mdn* = 3, 1.5–4) goal attainment for the Control group (*Z* = −.141, p = .888).

### Hypothesis 2: practicality

Average time to select and scale a goal was 10 min.

### Hypothesis 3: reliability and content validity

Table 3 shows that 10 of the 17 (59%) criteria proposed by Krasny-Pacini et al. (2016) were met in the current study. Two of four (50%) reliability of scale construction and two of five (40%) reliability of scale rating criteria were met. Three of four (75%) content validity criteria were met and three of four (75%) other criteria were met. The intraclass correlation coefficients of the GAS level ranges, which were calculated based on an absolute agreement, 2-way mixed effects model, for target goal 1 was .987, 95% CI (.974, .994), for target goal 2 was .986, 95% CI (.97, .994), for control goal 1 was 1, 95% CI (.999, 1), and for control goal 2 was .996, 95% CI (.993, .998), revealing excellent agreement.

**Table 3.** Krasny-Pacini et al. (2016) criteria met in the current study.

Domain	Criterion	Criterion met	Comment
Content validity			
	Collaborative goal setting	Yes	The first step of the GAS^b^ process involved giving the participant a choice of the goals they wanted to work on.
	Relevance/importance	No	Although selection of goals from the goal menu indicated relevance to the participant, an external judge was not asked to verify the relevance or clinical meaningfulness of the goals
	ICF^b^ classification of goal types	Yes	Documented in Results section
	Specificity	Yes	The 20 goals on the goal menu were related to the broad intervention target of improving executive functioning
Reliability of scale construction			
	Equidistance of levels	No	Equality of levels was not verified by an external judge
	Preintervention performance	Yes	All goals had their baseline set at the −2 GAS^a^ level.
	Attainability/difficulty	No	Although an external judge did not verify the attainability/difficulty of the scales, the examiner and participant collaboratively appraised the attainability of the scale in step 6b of the method. An external judge would have reduced examiner bias
	Time-specificity	Yes	Time 3 (follow up) GAS^a^ data were defined as the outcomes.
Reliability of scale rating			
	Interrater reliability	No	Outcomes were based on participant self-report
	Precise description of all levels	Yes	The calculator ensured all five GAS^a^ levels were clearly defined.
	Measurability	No	Outcomes were based on participant self-report
	Unidimensionality	Yes	The goal menu, calculator and overall method ensured only one variable was included per goal, and this was additionally confirmed by an external judge (first author)
	Context of measurement	No	Outcomes were based on participant self-report of their daily functioning
Other criteria			
	Training	Yes	Training was provided to the examiner, who was given the opportunity to practice GAS^a^ scaling with corrective feedback prior to the trial
	Examiner bias	No	The examiner was involved in both setting/scaling of the goals and post-intervention scoring
	Statistical analysis	Yes	Non-parametric analyses were used to analyse the data and calculate an effect size
	Provision of a sample scale	Yes	Included three examples of full GAS^a^ scales

^a^GAS = Goal attainment scale.

^b^ICF = International Classification of Functioning, Disability and Health.

All 46 control goals and 43 of 50 (86%) target goals belonged to the ICF Activities and Participation domain. Six (12%) target goals corresponded to the Body Functions domain due to a lack of specificity of the goals and one goal was not clear enough to be classified into an ICF domain. See Table 4 for examples of scaled GAS goals across three Activities and Participation ICF subdomains.

**Table 4.** Examples of GAS scale across three activities and participation ICF subdomains.

GAS^a^ Level	GAS^a^ Descriptor	Learning and applying knowledge goal	General tasks and demands goal	Self care goal
+2	Much better than expected	To concentrate for 56 to 60 minutes in groups	To wake up at 6:30am 6 to 7 times per week	To practice mindful eating during 17 to 21 meals per week
+1	Better than expected	To concentrate for 52 to 55 minutes in groups	To wake up at 6:30am 5 times per week	To practice mindful eating during 13 to 16 meals per week
0	Expected	To concentrate for 47 to 51 minutes in groups	To wake up at 6:30am 3 to 4 times per week	To practice mindful eating during 9 to 12 meals per week
−1	Less than expected	To concentrate for 43 to 46 minutes in groups	To wake up at 6:30am 2 times per week	To practice mindful eating during 5 to 8 meals per week
−2	Much less than expected	To concentrate for 38 to 42 minutes in groups	To wake up at 6:30am 0 to 1 times per week	To practice mindful eating during 0 to 4 meals per week

^a^GAS = Goal attainment scale.

### Hypothesis 4: construct validity

**Figure 1.**
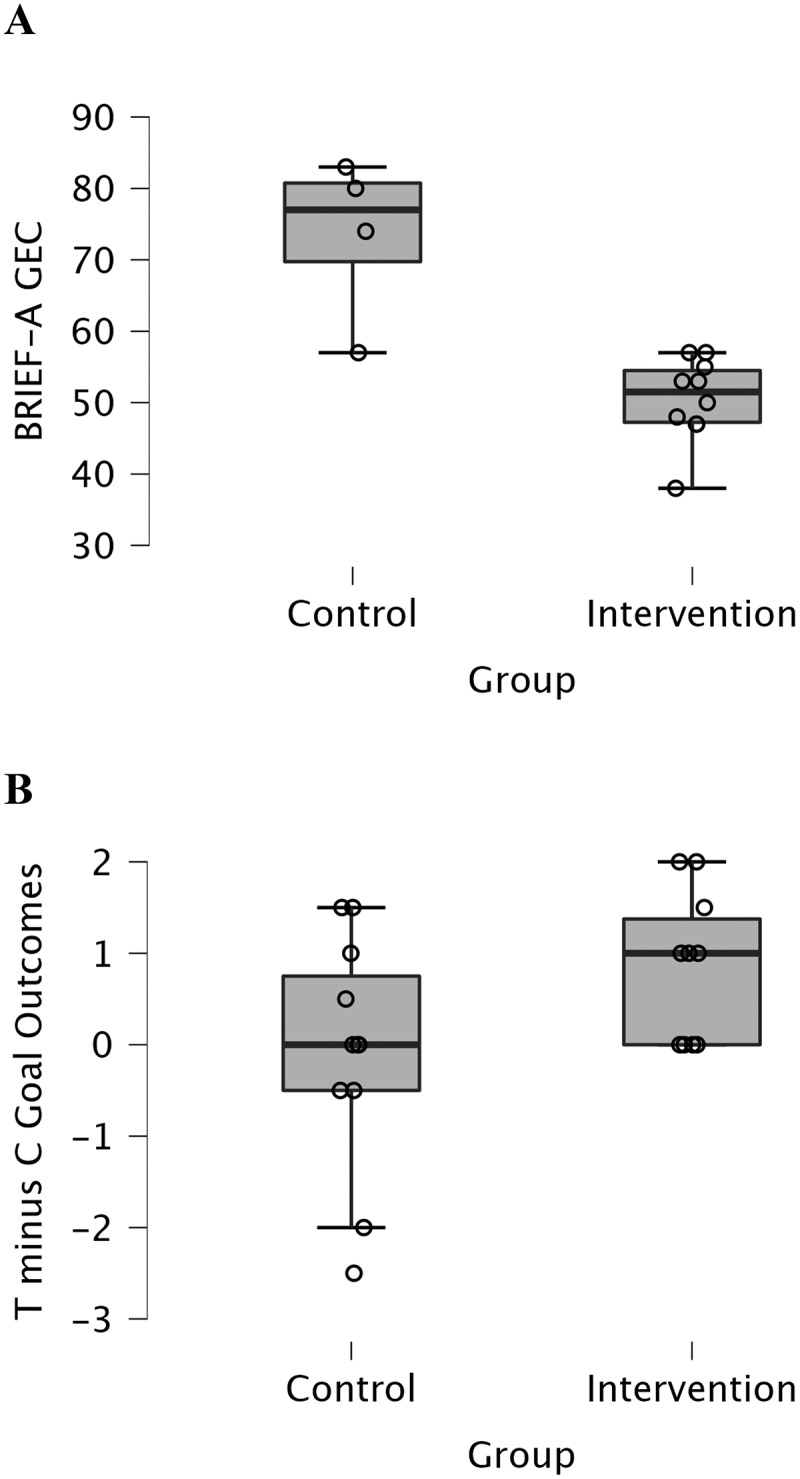
BRIEF-A GEC (panel A) and target minus control goal outcomes (panel B) across the control and intervention groups.

*Note*: BRIEF-A GEC = Behaviour Rating Inventory of Executive Function – Adult version Global Executive Composite; T = Target; C = Control.

Spearman rank order correlation between BRIEF-A GEC and target goals was −.455 (n = 14, p = .102). Spearman rank order correlation between BRIEF-A GEC and control goals was −.199 (n = 12, p = .535). Figure 1 shows BRIEF-A GEC (panel A) and Target minus Control goal outcomes (panel B) across groups.

## Discussion

The current study sought to examine the feasibility, reliability and validity of a modified approach to GAS in measuring goal attainment for residents of a drug and alcohol rehabilitation facility who were offered cognitive remediation. Consistent with the first hypothesis, there was significantly greater target than control goal attainment for the Intervention, but not the Control group, demonstrating limited efficacy. Furthermore, consistent with the second hypothesis, the modified GAS approach that made use of goal menus, an online calculator and control goals saved time, with an average duration of 10 min to set and scale a goal. This is much quicker than has been reported with conventional GAS (Grant & Ponsford, 2014; Grant et al., 2012). These efficiency gains constitute evidence for practicality. Together, these findings support feasibility of the novel approach.

Ten of the 17 (59%) criteria advanced by Krasny-Pacini et al. (2016) were met in the current study. However, only four of the nine (44%) reliability criteria were met. The items of interrater reliability, measurability and context of measurement were failed because the outcomes were based on participant self-report. The criterion of equidistance of levels stipulates that the difficulty from one level to the next should be verified by an external judge and that the levels should be *roughly* equal (Krasny-Pacini et al., 2016). The current study employed a statistical comparison of the ranges of the GAS levels across the two target and control goals, yielding exceptionally high levels of equivalence (>98%). However, because this was not verified by an external judge, this criterion was considered to have not been met. Future research may elucidate whether a statistical comparison as employed in this study might be sufficient to meet this criterion. Although attainability/difficulty was considered as part of the modified approach, this criterion was not met because it was not verified by an external judge.

Three of the four (75%) content validity items were met. The only item that was not met was relevance/importance, due to non-verification by an external judge. Three of the four (75%) other criteria were met, with the examiner bias item not being met due to the examiner being involved in both the goal setting and goal scoring phases.

The correlation between the BRIEF-A and target goal outcomes (convergent validity) was more than double that between the BRIEF-A and control goal outcomes (discriminant validity). Together, these findings provide evidence for construct validity of modified GAS outcomes with an inventory-based measure of executive functioning, a primary outcome measure of the Marceau et al. (2017) study. Indirectly, this also constitutes evidence of sensitivity or responsiveness of modified GAS.

Although goal menus have been noted to facilitate quicker generation of GAS goals (Turner-Stokes, 2009), the use of goal menus has been criticised for being contrary to the person-centred individualised approach of GAS (Grant & Ponsford, 2014; Playford et al., 2009). The benefits of a purely individualised approach without goal menus needs to be weighed against the costs of the added burden and time it takes to set highly individualised goals without the structure of a goal menu to facilitate this process. Use of the calculator ensured unidimensionality, a consideration of the range of all possible outcomes, the generation of ranges for all five GAS levels, and that there were no gaps or overshoots between the GAS levels, hence addressing many of the recommendations made by Grant and Ponsford (2014).

Use of control goals enabled the calculation of the effect of the intervention on target goals relative to control goals. The Pearson r effect size was .5, considered to be a medium effect (Cohen, 1988), whereby there is a 67% overlap between the curves for the two conditions (Zakzanis, 2001). This is one of the first studies to utilise non-parametric procedures to calculate an effect size for GAS as per the Krasny-Pacini et al. (2016) guidelines. There is practical utility in calculating such an effect size relative to a control condition given the criticism of GAS being too subjective to be used as an outcome measure in clinical trials (Tennant, 2007; Turner-Stokes, 2011). Given the effect pertains to the relative attainment of target compared with control goals, with each participant acting as their own control, the effect size is calculable even when there is no control group. This approach therefore lends itself to clinical research involving single cases and pre-post group studies. It may also be used as an adjunct to controlled group studies, which do not always find an effect across groups on a single GAS outcome measure (Herdman et al., 2018).

Control goal attainment for both groups was high, which indicated goal achievement at the “expected” and “better than expected” level on the GAS scale for the Intervention and Control groups, respectively. General non-specific factors associated with being a client of residential rehabilitation may partly explain this result. It is also possible that residential rehabilitation, particularly that offered in a therapeutic community, may directly target the types of ecological goals that were on the goal menu. The high attainment of target goals by the Control group may also be explained on the basis of the therapeutic nature of goal setting whereby the simple act of setting goals may itself result in greater goal attainment, even when there is no explicit striving towards the goal (Evans, 2012; Herdman et al., 2018). Regardless, the inclusion of control goals was able to control for any non-specific effects associated with the goal setting process.

The modified approach to goal setting adopted in this study differs from traditional GAS in that the “expected” outcome is calculated, rather than predicted. In traditional GAS, the goal setter is required to predict the goal outcome and then populate the other four levels of the GAS scale, whereas modified GAS requires the values for current level of functioning and maximum realistic level of functioning to calculate the ranges for the five GAS levels. The current approach involved informing participants that it was better to set goals at an intermediate point between these two levels rather than to strive for a stretch goal, which accords with the finding that people invest the highest level of effort in a task when it is perceived to be moderately difficult rather than when it is perceived as very easy or hard to achieve (Locke & Latham, 2002). This difference in defining the “expected” outcome level arguably represents a fundamental difference in the interpretation of the final GAS score across these approaches. With traditional GAS, the outcome represents both the goal setter’s goal attainment prediction skills and progress towards goals, whereas modified GAS outcomes represent progress towards the nominated goals independent of the goal setter’s prediction skills.

### Limitations and future directions

A limitation of the current study was that the Intervention group participants were provided with their target goals during an exercise in the latter part of the intervention, which likely inflated the effect size. Another limitation was the use of retrospective recall to determine goal outcomes, which is particularly unreliable in a population characterised by cognitive compromise. Similarly, retrospective estimation, rather than prospective recording, of the time taken to set and scale goals was used. There was insufficient power to conduct ordinal regression, which could have examined interaction effects between group and goal type to demonstrate within- and between-participant differences in goal outcomes. Finally, as there were no male participants, generalisation of findings to male residents of SUD rehabilitation is limited.

Sources of bias evident in the current study could be addressed in future research by asking participants to rate the relevance and importance of goal attainment, having the clinical meaningfulness and attainability of the goals rated by an external judge, and having an independent rater measure post-intervention goal attainment. It is also recommended that data be collected prospectively by both the participants and informants and/or have independent raters rate video recordings of the behaviours relevant to the goal outcomes during contrived assessment tasks or in ecological settings.

Another consideration for future research is to develop a repository of goals based on the ICF codes, with associated maximum realistic level and current functioning questions and make this available to researchers and clinicians to facilitate like-by-like comparisons across studies. This would also allow for the tracking of goal type choice by various clinical populations, which could aid in the generation of appropriate goal menus for use with particular clinical groups.

## Conclusion

The present study addresses a gap in the neuropsychological intervention literature by describing a novel process of measuring individualised, person-centred goal outcomes to supplement the results of standardised performance- and inventory-based measures that are typically used as outcome measures in cognitive intervention evaluation studies (Cicerone et al., 2000, 2005, 2011, 2019). The present modified approach to GAS met the Bowen et al. (2009) feasibility criteria of limited efficacy (i.e., an effect size of target to control goals was calculated), and practicality (i.e., efficiency of goal identification and scaling). Although content and construct validity were demonstrated, fewer than half of the reliability criteria advanced by Krasny-Pacini et al. (2016) were met, requiring further refinement of and research into this novel approach to GAS.

## Supplementary Material

Supplemental Material

## Data Availability

The data that support the findings of this study are available from the corresponding author upon reasonable request https://data.mendeley.com/datasets/3w3rb3stt2/1.

## References

[cit0001] Bowen, D. J., Kreuter, M., Spring, B., Cofta-Woerpel, L., Linnan, L., Weiner, D., Bakken, S., Kaplan, C. P., Squiers, L., Fabrizio, C., & Fernandez, M. (2009). How we design feasibility studies. *American Journal of Preventive Medicine*, 36(5), 452–14. 10.1016/j.amepre.2009.02.002 19362699 PMC2859314

[cit0002] Brown, D. A., Effgen, S. K., & Palisano, R. J. (1998). Performance following ability-focused physical therapy intervention in individuals with severely limited physical and cognitive abilities. *Physical Therapy*, 78(9), 934–947. 10.1093/ptj/78.9.934 9736892

[cit0003] Cicerone, K. D., Dahlberg, C., Kalmar, K., Langenbahn, D. M., Malec, J. F., Bergquist, T. F., Felicetti, T., Giacino, J. T., Harley, J. P., Harrington, D. E., Herzog, J., Kneipp, S., Laatsch, L., & Morse, P. A. (2000). Evidence-based cognitive rehabilitation: Recommendations for clinical practice. *Archives of Physical Medicine and Rehabilitation*, 81(December), 1596–1615. 10.1053/apmr.2000.19240 11128897

[cit0004] Cicerone, K. D., Dahlberg, C., Malec, J. F., Langenbahn, D. M., Felicetti, T., Kneipp, S., Ellmo, W., Kalmar, K., Giacino, J. T., Harley, J. P., Laatsch, L., Morse, P. A., & Catanese, J. (2005). Evidence-based cognitive rehabilitation: Updated review of the literature from 1998 through 2002. *Archives of Physical Medicine and Rehabilitation*, 86(8), 1681–1692. 10.1016/j.apmr.2005.03.024 16084827

[cit0005] Cicerone, K. D., Goldin, Y., Ganci, K., Rosenbaum, A., Wethe, J. V., Langenbahn, D. M., Malec, J. F., Bergquist, T. F., Kingsley, K., Nagele, D., Trexler, L., Fraas, M., Bogdanova, Y., & Harley, J. P. (2019). Evidence-based cognitive rehabilitation: Systematic review of the literature from 2009 through 2014. *Archives of Physical Medicine and Rehabilitation*, 100(8), 1515–1533. 10.1016/j.apmr.2019.02.011 30926291

[cit0006] Cicerone, K. D., Langenbahn, D. M., Braden, C., Malec, J. F., Kalmar, K., Fraas, M., Felicetti, T., Laatsch, L., Harley, J. P., Bergquist, T., Azulay, J., Cantor, J., & Ashman, T. (2011). Evidence-based cognitive rehabilitation: Updated review of the literature from 2003 through 2008. *Archives of Physical Medicine and Rehabilitation*, 92(4), 519–530. 10.1016/j.apmr.2010.11.015 21440699

[cit0007] Clark, M., Miller, A., Berry, J., & Cheng, K. (2021). Mental contrasting with implementation intentions increases study time for university students. *The British Journal of Educational Psychology*, 91(3), 850–864. 10.1111/bjep.12396 33315247

[cit0008] Cohen, J. (1988). *Statistical power analysis for the behavioral sciences*. Erlbaum Associates.

[cit0009] Ertzgaard, P., Ward, A. B., Wissel, J., & Borg, J. (2011). Practical considerations for goal attainment scaling during rehabilitation following acquired brain injury. *Journal of Rehabilitation Medicine*, 43(1), 8–14. 10.2340/16501977-0664 21174050

[cit0010] Evans, J. J. (2012). Goal setting during rehabilitation early and late after acquired brain injury. *Current Opinion in Neurology*, 25(6), 651–655. 10.1097/WCO.0b013e3283598f75 23007008

[cit0011] Fernández-Serrano, M. J., Pérez-García, M., Schmidt Río-Valle, J., & Verdejo-García, A. (2010). Neuropsychological consequences of alcohol and drug abuse on different components of executive functions. *Journal of Psychopharmacology*, 24(9), 1317–1332. 10.1177/0269881109349841 20007413

[cit0012] Fritz, C. O., Morris, P. E., & Richler, J. J. (2012). Effect size estimates: Current use, calculations, and interpretation. *Journal of Experimental Psychology General*, 141(1), 2–18. 10.1037/a0024338 21823805

[cit0013] Golden, C., & Freshwater, S. M. (2002). *Stroop color and word test adult version: A manual for clinical and experimental uses*. Nova Southeastern University: Stoelting.

[cit0014] Grant, M., & Ponsford, J. (2014). Goal attainment scaling in brain injury rehabilitation: Strengths, limitations and recommendations for future applications. *Neuropsychological Rehabilitation*, 24(December), 661–677. 10.1080/09602011.2014.901228 24787703

[cit0015] Grant, M., Ponsford, J., & Bennett, P. C. (2012). The application of goal management training to aspects of financial management in individuals with traumatic brain injury. *Neuropsychological Rehabilitation*, 22(6), 1–22. 10.1080/09602011.2012.693455 22812612

[cit0016] Herdman, K. A., Davidson, S., Au, A., Davidson, S., Vandermorris, S., Vandermorris, S., Davidson, S., Au, A., Troyer, A. K., & Herdman, K. A. (2018). Comparable achievement of client-identified, self- rated goals in intervention and no-intervention groups: Reevaluating the use of goal attainment scaling as an outcome measure. *Neuropsychological Rehabilitation*, 29(10), 1–11. 10.1080/09602011.2018.1432490 29430998

[cit0017] Kiresuk, T. J., & Sherman, R. E. (1968). Goal attainment scaling: A general method for evaluating comprehensive community mental health programs. *Community Mental Health Journal*, 4(6), 443–453. 10.1007/BF01530764 24185570

[cit0018] Krasny-Pacini, A., Evans, J., Sohlberg, M., & Chevignard, M. (2016). Proposed criteria for appraising goal attainment scales used as outcome measures in rehabilitation research. *Archives of Physical Medicine and Rehabilitation*, 97(1), 157–170. 10.1016/j.apmr.2015.08.424 26343173

[cit0019] Levine, B., Schweizer, T. A., O’connor, C., Turner, G., Gillingham, S., Stuss, D. T., Manly, T., & Robertson, I. H. (2011). Rehabilitation of executive functioning in patients with frontal lobe brain damage with goal management training. *Frontiers in human neuroscience*, 5(9), 1–9. 10.3389/fnhum.2011.00009 21369362 PMC3043269

[cit0020] Little, R. J. (1988). A test of missing completely at random for multivariate data with missing values. *Journal of the American Statistical Association*, 83(404), 1198–1202. 10.1080/01621459.1988.10478722

[cit0021] Locke, E. A., & Latham, G. P. (2002). Building a practically useful theory of goal setting and task motivation. A 35-year odyssey. *The American Psychologist*, 57(9), 705–717. 10.1037/0003-066X.57.9.705 12237980

[cit0022] Lumosity. (2021). www.lumosity.com

[cit0023] MacKay, G., Somerville, W., & Lundie, J. (1996). Reflections on goal attainment scaling (GAS): Cautionary notes and proposals for development. *Educational Research*, 38(2), 161–172. 10.1080/0013188960380204

[cit0024] Malec, J. F. (1999). Goal attainment scaling in rehabilitation. *Neuropsychological Rehabilitation*, 9(March 15), 253–275. 10.1080/096020199389365

[cit0025] Maloney, P. W., Grawitch, M. J., & Barber, L. K. (2012). The multi-factor structure of the brief self-control scale: Discriminant validity of restraint and impulsivity. *Journal of Research in Personality*, 46(1), 111–115. 10.1016/j.jrp.2011.10.001

[cit0026] Marceau, E. M., Berry, J., Lunn, J., Kelly, P. J., & Solowij, N. (2017). Cognitive remediation improves executive functions, self-regulation and quality of life in residents of a substance use disorder therapeutic community. *Drug and alcohol dependence*, 178(June), 150–158. 10.1016/j.drugalcdep.2017.04.023 28651150

[cit0027] Marceau, E. M., Lunn, J., Berry, J., Kelly, P. J., & Solowij, N. (2016). The Montreal Cognitive Assessment (MoCA) is sensitive to head injury and cognitive impairment in a residential alcohol and other drug therapeutic community. *Journal of Substance Abuse Treatment*, 66, 30–36. 10.1016/j.jsat.2016.03.002 27211994

[cit0028] Nardo, T., Batchelor, J., Berry, J., Francis, H., Jafar, D., & Borchard, T. (2022). Cognitive Remediation as an Adjunct Treatment for Substance Use Disorders: A Systematic Review. *Neuropsychology Review*, 32(1), 161–191. 10.1007/s11065-021-09506-3 33871785

[cit0029] Pallant, J. (2016). *SPSS survival manual: A step by step guide to data analysis using SPSS program* (6th ed.). McGraw-Hill Education.

[cit0030] Patton, J. H., Stanford, M. S., & Barratt, E. S. (1995). Factor structure of the Barratt impulsiveness scale. *Journal of Clinical Psychology*, 51(6), 768–774. 10.1002/1097-4679(199511)51:6<768:AID-JCLP2270510607>3.0.CO;2-1 8778124

[cit0031] Pearson Assessment. (2009) . *Advanced clinical solutions for WAIS-IV and WMS-IV: Clinical and interpretive manual*. NCS Pearson Inc.

[cit0032] Playford, E. D., Siegert, R., Levack, W., & Freeman, J. (2009). Areas of consensus and controversy about goal setting in rehabilitation: A conference report. *Clinical Rehabilitation*, 23(4), 334–344. 10.1177/0269215509103506 19449469

[cit0033] Ponsford, J., Bayley, M., Wiseman-Hakes, C., Togher, L., Velikonja, D., McIntyre, A., Janzen, S., & Tate, R. (2014). INCOG recommendations for management of cognition following traumatic brain injury, part II: Attention and information processing speed. *The Journal of Head Trauma Rehabilitation*, 29(4), 321–337. 10.1097/HTR.0000000000000072 24984095

[cit0034] Roth, R., Isquith, P., & Gioia, G. (2005). *Behavior Rating Inventory of Executive Functioning - Adult version (BRIEF-A)*. PAR.

[cit0035] Ruble, L., McGrew, J. H., & Toland, M. D. (2012). Goal attainment scaling as an outcome measure in randomized controlled trials of psychosocial interventions in autism. *Journal of Autism and Developmental Disorders*, 42(9), 1974–1983. 10.1007/s10803-012-1446-7 22271197 PMC3358457

[cit0036] Schlosser, R. W. (2004). Goal attainment scaling as a clinical measurement technique in communication disorders: A critical review. *Journal of Communication Disorders*, 37(3), 217–239. 10.1016/j.jcomdis.2003.09.003 15063144

[cit0037] Sheehan, D. V., Lecrubier, Y., Sheehan, K. H., Amorim, P., Janavs, J., Weiller, E., Hergueta, T., Baker, R., & Dunbar, G. C. (1998). The Mini-International Neuropsychiatric Interview (M.I.N.I): The development and validation of a structured diagnostic psychiatric interview for DSM-IV and ICD-10. *Journal Clinica Psychiatry*, 59(Suppl. 20), 22–33. 9881538

[cit0038] Steenbeek, D., Ketelaar, M., Galama, K., & Gorter, J. W. (2007). Goal attainment scaling in paediatric rehabilitation: A critical review of the literature. *Developmental Medicine and Child Neurology*, 49(7), 550–556. 10.1111/j.1469-8749.2007.00550.x 17593130

[cit0039] Tate, R., Kennedy, M., Ponsford, J., Douglas, J., Velikonja, D., Bayley, M., & Stergiou-Kita, M. (2014). INCOG recommendations for management of cognition following traumatic brain injury, part III: Executive function and self-awareness. *The Journal of Head Trauma Rehabilitation*, 29(4), 338–352. 10.1097/HTR.0000000000000068 24984096

[cit0040] Tennant, A. (2007). Goal attainment scaling: Current methodological challenges. *Disability and Rehabilitation*, 29(20–21), 1583–1588. 10.1080/09638280701618828 17882728

[cit0041] Togher, L., Wiseman-Hakes, C., Douglas, J., Stergiou-Kita, M., Ponsford, J., Teasell, R., Bayley, M., & Turkstra, L. S. (2014). INCOG recommendations for management of cognition following traumatic brain injury, part IV: Cognitive communication. *The Journal of Head Trauma Rehabilitation*, 29(4), 353–368. 10.1097/HTR.0000000000000071 24984097

[cit0042] Turner-Stokes, L. (2009). Goal attainment scaling (GAS) in rehabilitation: A practical guide. *Clinical Rehabilitation*, 23(4), 362–370. 10.1177/0269215508101742 19179355

[cit0043] Turner-Stokes, L. (2011). Goal attainment scaling and its relationship with standardized outcome measures: A commentary. *Journal of Rehabilitation Medicine*, 43(1), 70–72. 10.2340/16501977-0656 21174054

[cit0044] Turner-Stokes, L., Baguley, I. J., De Graaff, S., Katrak, P., Davies, L., McCrory, P., & Hughes, A. (2010). Goal attainment scaling in the evaluation of treatment of upper limb spasticity with botulinum toxin: A secondary analysis from a double-blind placebo-controlled randomized clinical trial. *Journal of Rehabilitation Medicine*, 42(1), 81–89. 10.2340/16501977-0474 20111849

[cit0045] Valls-Serrano, C., Verdejo-García, A., & Caracuel, A. (2016). Planning deficits in polysubstance dependent users: Differential associations with severity of drug use and intelligence. *Drug and Alcohol Dependence*, 162, 72–78. 10.1016/j.drugalcdep.2016.02.027 26971229

[cit0046] Velikonja, D., Tate, R., Ponsford, J., Mcintyre, A., Janzen, S., & Bayley, M. (2014). INCOG recommendations for management of cognition following traumatic brain injury, part V: Memory. *The Journal of Head Trauma Rehabilitation*, 29(4), 369–386. 10.1097/HTR.0000000000000069 24984098

[cit0047] WHO. (2002). *Towards a common language for functioning, disability and health ICF* (Vol. 1149). https://WHO/EIP/GPE/CAS/01.3

[cit0048] Zakzanis, K. K. (2001). Statistics to tell the truth, the whole truth, and nothing but the truth. Formulae, illustrative numerical examples, and heuristic interpretation of effect size analyses for neuropsychological researchers. *Archives of Clinical Neuropsychology*, 16(7), 653–667. 10.1093/arclin/16.7.653 14589784

