# Supplementary material for: Feasibility, reliability and validity of a modified approach to goal attainment scaling to measure goal outcomes following cognitive remediation in a residential substance use disorder rehabilitation setting
Source: Aust J Psychol. 2023 Feb 19;75(1):2170652. doi: 10.1080/00049530.2023.2170652 (PMC12175606; doi:10.1080/00049530.2023.2170652)
Supplement: Supplemental Material [file RAUP_A_2170652_SM2134.docx]

**Supplement 1 – Goal Menu**

1. To eat healthier food
2. To spend money more thoughtfully (not impulsively)
3. To spend money within budget (not to overspend)
4. To control my temper or emotions
5. To use time more productively
6. To be punctual
7. To do things right away
8. To maintain dental hygiene
9. To wake up on time
10. To maintain a tidy living area
11. To be able to find things quickly and easily
12. To see things through to completion
13. To make a start on complex or big tasks
14. To concentrate better whilst __________
15. To remember __________
16. To get ready for the day
17. To start things well ahead of deadlines
18. To think ahead of consequences before acting
19. To respond better to change
20. To be more patient and take my time

**Supplement 2 – Maximum Realistic Level and Current Functioning Questions**

MRL = maximum realistic level CF = current functioning

| 1. To eat healthier food | |
| --- | --- |
|  | MRL: In a typical day / week / fortnight (circle one), how many meals do you have? |
|  | CF: How many healthy meals do you currently have per day / week / fortnight (circle one) |
| 2. To spend money more thoughtfully (not impulsively) | |
|  | MRL: In a typical day / week / fortnight (circle one), how many times do you make a purchase? |
|  | CF: How many impulsive purchases do you currently make per day / week / fortnight (circle one) |
| 3. To spend money within budget (not to overspend) | |
|  | MRL: In a typical day / week / fortnight (circle one), how many times do you make a purchase? |
|  | CF: How many overspending purchases do you currently make per day / week / fortnight (circle one) |
| 4. To control my temper or emotions | |
|  | MRL: In a typical day / week / fortnight (circle one), how many times do you get upset or emotional? |
|  | CF: How many times are you currently able to control your temper or emotions per day / week / fortnight (circle one) |
| 5. To use time more productively | |
|  | MRL: In a typical day / week / fortnight (circle one), how much free time do you have (in hours)? |
|  | CF: How many of your free time hours are currently used productively per day / week / fortnight (circle one) |
| 6. To be punctual | |
|  | MRL: In a typical day / week / fortnight (circle one), how often do you need to be punctual? |
|  | CF: How many times per day / week / fortnight (circle one) are you currently punctual |
| 7. To do things right away | |
|  | MRL: In a typical day / week / fortnight (circle one), how many times do you need to do a task that you could possibly postpone? |
|  | CF: How many times per day / week / fortnight (circle one) do you currently do something right away |
| 8. To maintain dental hygiene | |
|  | MRL: In a typical day / week / fortnight (circle one), how many times do most people brush (or floss) their teeth? |
|  | CF: How many times per day / week / fortnight (circle one) do you currently brush (or floss) your teeth? |
| 9. To wake up on time | |
|  | MRL: In a typical day / week / fortnight (circle one), how many times do you need to wake up by __am? (first establish a reasonable waking time) |
|  | CF: How many times per day / week / fortnight (circle one) do you currently wake up by __am? |
| 10. To maintain a tidy living area | |
|  | MRL: In a typical day / week / fortnight (circle one), how many times do most people tidy up their living area? |
|  | CF: How many times per day / week / fortnight (circle one) do you currently tidy up your living area? |

| 11. To be able to find things quickly and easily | |
| --- | --- |
|  | MRL: In a typical day / week / fortnight (circle one), how many times do you need to find things quickly and easily? |
|  | CF: How many times per day / week / fortnight (circle one) are you currently able to find things quickly and easily? |
| 12. To see things through to completion | |
|  | MRL: In a typical day / week / fortnight (circle one), how many times do you need to see a task through to completion? |
|  | CF: How many times per day / week / fortnight (circle one) do you currently see things through to completion? |
| 13. To make a start on complex or big tasks | |
|  | MRL: In a typical day / week / fortnight (circle one), how many times do you need to start a complex or big task? |
|  | CF: How many times per day / week / fortnight (circle one) do you currently start complex or big tasks? |
| 14. To concentrate better whilst __________ | |
|  | MRL: In a typical day / week / fortnight (circle one), how many times do you need to concentrate for ___ minutes whist _______? (first establish appropriate concentration span for the activity) |
|  | CF: How many times per day / week / fortnight (circle one) are you currently able to concentrate for ___ minutes whilst ________? |
| 15. To remember __________ | |
|  | MRL: In a typical day / week / fortnight (circle one), how many times do you need to remember ________? |
|  | CF: How many times per day / week / fortnight (circle one) do you currently remember ________? |

| 16. To get ready for the day | |
| --- | --- |
|  | MRL: In a typical day / week / fortnight (circle one), how many times do you need to prepare for the day? |
|  | CF: How many times per day / week / fortnight (circle one) do you currently prepare for the day? |
| 17. To start things well ahead of deadlines | |
|  | MRL: In a typical day / week / fortnight (circle one), how many times do you need to start things well ahead of a deadline? |
|  | CF: How many times per day / week / fortnight (circle one) do you currently start things well ahead of a deadline? |
| 18. To think ahead of consequences before acting | |
|  | MRL: In a typical day / week / fortnight (circle one), how many times do you need to think of consequences before you act? |
|  | CF: How many times per day / week / fortnight (circle one) do you currently think of consequences before you act? |
| 19. To respond better to change | |
|  | MRL: In a typical day / week / fortnight (circle one), how many times do you experience unexpected changes? |
|  | CF: How many times per day / week / fortnight (circle one) do you currently respond well to changes? (need to define “responding well”) |
| 20. To be more patient and take my time | |
|  | MRL: In a typical day / week / fortnight (circle one), how many times do you need to slow down and take your time? |
|  | CF: How many times per day / week / fortnight (circle one) do you currently slow down and take your time? |
